# Comparison between the impact of osmotic and NaCl treatments on the expression of genes coding for ion transporters in *Oryza glaberrima* Steud.

**DOI:** 10.1371/journal.pone.0290752

**Published:** 2023-11-15

**Authors:** Hermann Prodjinoto, Willy Irakoze, Christophe Gandonou, Muriel Quinet, Stanley Lutts

**Affiliations:** 1 Groupe de Recherche en Physiologie végétale – Earth and Life Institute-Agronomy (ELIA) – Université catholique de Louvain, Louvain-la-Neuve, Belgium; 2 Laboratoire de Physiologie végétale et d’Etude des Stress environnementaux, Faculté des Sciences et Techniques, Université d’Abomey-Calavi, Cotonou, République du Bénin; 3 Faculté d’Agronomie et de Bio-ingénierie, Université du Burundi, Bujumbura, Burundi; Central University of Haryana School of Life Sciences, INDIA

## Abstract

We analyzed the expression of genes coding for Na^+^ transporters (*OsHKT1*.*5*, *OsHKT1*.*1*, *OsSOS1*, *OsSOS2*, *OsNHX1*, *OsNHX2*), Cl^-^ transporter (*OsNRT1*, *OsCLC*, *OsCCC1*) and gene coding for the transcription factor DREB (*OsDREB2*) involved in response to desiccation in two cultivars of *O*. *glaberrrima* differing in salt-resistance (salt-tolerant cultivar (TOG5307) and salt-sensitive (TOG 5949)) exposed to NaCl, PEG or both agents present simultaneously. Seedlings were grown in iso-osmotic nutrient solution (*Ψ*s = -0.47±0.02 MPa) containing PEG 6,000 12.9% (water stress), NaCl 75 mM (salt stress) and PEG 6.4% + NaCl 37.5 mM (MIX-treatment) during 1 and 7 days. Plants were analyzed for gene expression, mineral nutrients, and photosynthetic-related parameters. Na^+^ and Cl^-^ accumulations in salt-treated plants were lower in roots and shoots of TOG5307 comparatively to TOG5949 while water content decreased in TOG5307. TOG5307 exhibited tolerance to water stress and maintained higher net photosynthesis and water use efficiency than TOG5949 in response to all treatments, but was less efficient for osmotic adjustment. Dehydration tolerance of TOG5307 involves a higher *OsDREB2* expression. TOG5307 also exhibited a higher *OsSOS1*, *OsSOS2*, *OsNHX1* and *OsNHX2* expression than TOG5949 in response to salinity. *OsHKT1*.*5* was slightly induced in the shoot. *OsHKT1*.*1* was recorded in the shoots but remained undetectable in the roots. Chloride and sodium accumulations were strongly reduced in the shoots when PEG was present. Salinity resistance in *Oryza glaberrima* implies tolerance to dehydration as well as complementary strategies of Na^+^ exclusion through the SOS system and Na^+^ tolerance through vacuolar sequestration.

## Introduction

Salinity is a major environmental constraint hampering plant growth and development. It has a major deleterious impact on cultivated plant yields and compromises food security in numerous areas of the world [1]. An excess of soluble salts in the environment may act on almost all aspects of plant physiology: it compromises the plant water status, affects plant mineral nutrition and photosynthesis, modifies the hormonal profile and induces oxidative stress [2]. It mainly acts through an osmotic component related to a decrease in the external water potential and through an ionic component linked to the progressive accumulation of toxic ions in plant tissues [3]. Nevertheless, the respective importance of the two types of constraints is still a matter of debate [1, 2]. Any attempt of generalization should however be considered with caution. The relative importance of ionic and osmotic components depends on the plant species, the used NaCl dose, the plant developmental stage and the kinetics of salt application [4, 5]. Moreover, the osmotic and ionic components should not necessarily be regarded as independent additive constraints even if they act on similar properties through distinct mechanisms. Plant species are unable tolerate a high salt concentration in their cytosol since Na^+^ and Cl^-^ excess are highly toxic to cytosolic enzymes. It has however been hypothesized that on a short-term basis, ions may help the plants to perform osmotic adjustment before they acquire the capacity to synthesize enough osmocompatible solutes like proline or soluble sugars [6–8].

Ion absorption and distribution are regulated by a complex network of membrane transporters, channels and pumps mediating Na^+^ and K^+^ homeostasis. Na^+^ enters via non selective cation channels (NSCC) and possibly via other cation transporters. The Na^+^/H^+^ antiporter SOS1 extrudes Na^+^ at the root soil interface [8–10] but is also reported to be involved in Na^+^ unloading at the xylem as the corresponding gene is expressed on xylem/parenchyma cell interface. SOS1 itself is regulated in close interaction with SOS2 and SOS3 proteins and the SOS system plays a key role in salt tolerance in several angiosperm plant species [11]. The vacuolar sequestration of Na^+^ is under the control of NHX transporters which mediate the electroneutral Na^+^/H^+^ exchange, driving the excess of cytosolic Na^+^ into the vacuole [12]. Numerous NHX exist in plant species and their functions cannot be solely explained by Na^+^ accumulation in vacuoles [13]. Other transporters called HKT are involved in Na^+^ unloading from the xylem vessels. This transporter family may act not only at the xylem loading site itself, but may also transfer Na^+^ from the xylem to the phloem, thus avoiding Na^+^ accumulation in aerial parts through Na^+^ cycling [14].

Chloride accumulation received far less attention than Na^+^ content in studies devoted to salt tolerance in plants. Chloride is an essential element required for photosynthesis, acts as a counter anion to stabilize membrane potential and is involved in turgor and pH regulation but becomes rapidly toxic if accumulated at high concentrations [15–17]. Numerous transporters are involved in Cl^-^ movement across plant membranes. The CLC Chloride Channel family specifically transport chloride as Cl^-^/H^+^ antiporters and is also reported to be involved in vacuolar nitrate transport [18]. Other nitrate transporters contribute to Cl^-^ uptake in response to high salinity [19, 20]. Finally, the Cation-Chloride-Cotransporter is a membrane-integral solute carrier able to mediate electroneutral translocation of Cl^-^ coupled to K^+^ and/or Na^+^ [21].

Rice (*Oryza sativa* L.) is a very salt-sensitive species, mainly at the seedling and at the flowering stages [22, 23]. Rice is among the most important plant for human nutrition and considering the global increasing population it is now well accepted that its culture should be extended to marginal lands which are not currently suitable for rice production. A major portion of these lands are affected by drought and/or by salinity so that the improvement of salinity resistance in this species appears as a priority [24, 25] Beside the cultivated rice species *Oryza sativa* L, wild relative may provide useful genes to improve salinity resistance but interspecific crosses and cultivar selection is a long-term task and require a big deal of work [23, 26, 27]. *Oryza glaberrima* Steud. is an African rice species which is marginally cultivated in Africa but exhibits interesting abilities to cope with environmental constraints [28, 29]. Although some informations are currently available for its adaptation to soil drying [30, 31] or to iron toxicity [32, 33], its level of resistance to salinity remains poorly documented and requires additional investigations. Ren et al. [34] showed that *OsHKT1;5* is involved in the salt tolerance of *O*. *sativa* cv Nona Bokra and codes for a plasma membrane transporter. Platten et al. [35] demonstrated that *HKT1;5* also plays a key role in *O*. *glaberrima* and contributes to reduce Na^+^ concentration in the leaf blades of the African rice species. Prodjinoto et al. [36] demonstrated that Na^+^ is more toxic than Cl^-^ for the African rice species. The pattern of modification induced by both types of ions was similar but the quantitative aspects of the response strongly differ between a salt-tolerant cv TOG 5307 and a salt-sensitive one (TOG 5949). In an additional study, Prodjinoto et al. [37] used inhibitors of Na^+^-H^+^ antiporter (amiloride 100 μM) and cation-chloride-cotransporter (CCC) (bumetanide 200 μM): the salt-tolerant cv TOG5307 regulated the Na^+^ uptake more efficiently than the sensitive cv while bumetanide reduced Cl^-^ accumulation in both cultivars, although the accompanying cation may differ between them.

Most Na^+^ and Cl^-^ transporters were never studied in *O*. *glaberrima*. Such a paucity of information prompted us to analyze the expression of genes coding for Na^+^ transporters (*OsHKT1*.*5*, *OsHKT1*.*1*, *OsSOS1*, *OsSOS2*, Os*NHX1*, *OsNHX2*), Cl^-^ transporter (*OsNRT1*, *OsCLC*, *OsCCC1*) and one gene coding for the transcription factor DREB2 in two cultivars of *O*. *glaberrrima* differing in salt tolerance and exposed to iso-osmotic solutions containing NaCl, PEG or both agents present simultaneously (NaCl + PEG). Plants were exposed to stress for 1 and 7 days. Gene expressions were analyzed in relation to physiological parameters related to plant growth, mineral nutrition, plant water status and photosynthetic-related parameters.

## Material and methods

### Plant material and growing conditions

Seeds of African rice cultivars TOG5307 (salt-tolerant, AccNumber WAB0021855) and TOG5949 (salt-sensitive; AccNumber WAB0020144) were obtained from AfricaRice (Bouaké, Ivory coast). Non-dehusked seeds were germinated on two layers of Whatman papers (85 mm, Grade 1) moistened with sterile deionized water in a growth chamber at 25 °C and under a 12 h daylight period. Ten-days-old equally grown seedlings were transferred to hydroponic culture system into phytotron at 29/26 °C under 16/8 h day/night period. Light intensity was 300 μmol m^-2^ s^-1^ (HPIT/400W from PHILIPS metal iodide lamp) and relative humidity was 70 ± 5%. Seedlings were fixed on polystyrene plates floating on 16 L of Yoshida nutritive solution [38]. After 7 days of acclimatization in control conditions, the seedlings were randomly divided into four groups:(1) control: plants grown in Yoshida nutritive solution (EC = 0.71±0.009 mS.cm^-1^; *Ψ*s = -0.08±0.003 MPa), (2) PEG: plants grown in Yoshida nutritive solution + 12.9% of PEG 6,000 (EC = 0.73±0.003 mS.cm^-1^; *Ψ*s = -0.47±0.02 MPa), (3) NaCl: plants grown in Yoshida nutritive solution containing 75 mM NaCl, (EC = 7.72±0.08 mS.cm^-1^; *Ψ*s = -0.46±0.02 MPa) and (4) MIX treatment: plants grown in Yoshida nutritive solution containing 37.5 mM NaCl + 6.4% of PEG 6,000 (EC = 4.10±0.02 mS.cm^-1^; *Ψ*s = -0.45±0.06 MPa). This implies that all stressing solutions had the same osmotic potential but exhibited different electrical conductivities. The second actively growing leaf (numbering from the top of the plant) present at the time of treatment application was tagged for subsequent physiological measurements. After 1 day and 7 days of treatment, the plants were harvested and divided into shoots and roots for biochemical and molecular analysis. Roots were rinsed for 30 s in deionized water to remove ions from the free spaces.

### Plant growth, water content, osmotic potential, and ions determination

Plant growth was determined on the basis of the relative increase in leaf number and the relative increase in root and shoot length established considering the formula [(X_f_ − X_i_) /X_i_], where X_f_ and X_i_ are the final and initial X measurement. Shoot and root fresh weight (FW) and dry weight (DW) were estimated on six individual plants per treatment. For DW determination, roots and shoots were incubated in an oven at 70 °C for 72 h until reaching constant dry weight. Water content (WC) was calculated as WC = (FW–DW)/FW * 100.

Osmotic potential (*Ψ*s) was estimated on the extracted sap using a Wescor 5600 vapor pressure osmometer as previously described [39]. For the determination of Cl^-^ concentration, 20 mg DW were digested with nitric acid (0.5%) and the mixture was stirred for 48 hours. The supernatant recovered after centrifugation at 10,000 *g* for 10 min at 25 °C was subsequently filtered with Wathman n° 2 paper and then quantified via liquid chromatography (HPLC-Dionex ICS2000). For Na^+^ and K^+^ quantification, 50 mg DW were incubated in 4mL of 35% HNO_3_ at 80 °C. The residue was redissolved with aqua regia (HCl 37%: HNO_3_ 65%, 3:1) and filtered with Whatman n° 2 paper. Elements were quantified by flame atomic absorption spectrophotometry (ICE 3300; Thermo Scientific, Waltham, MA). All measurements were performed using technical triplicates per sample.

### Gas-exchange parameters, chlorophyll-fluorescence and stomatal conductance

The second actively growing leaf (numbering from the top of the plant) was used for photosynthesis-related parameters estimation. The instantaneous CO_2_ assimilation under ambient conditions (400 ppm CO_2_) (*A*), the instantaneous transpiration (*E*) and inter-cellular CO_2_ concentration (*C*i) were estimated using an Infrared Gas Analyzer (LCA4 8.7 ADC, Bioscience, Hertfordshire, UK). The gas exchange was measured using a Parkinson leaf cuvette, on intact leaves for 1 min (20 records min^−1^) with an air flow of 300 mL min^−1^ under ambient light conditions. Chlorophyll fluorescence was determined using a portable pulse-modulated chlorophyll fluorimeter (FMS2, Hansatech, King’s Lynn, UK). The actual PSII efficiency (Ф_PSII_), the photochemical quenching coefficient (q_P_), the non-photochemical quenching (NPQ) and the maximal efficiency of PSII photochemistry in the dark-adapted state (*F*_v_/*F*_m_) were assessed as previously described [40]. Leaf stomatal conductance (*g*_s_) was measured using an AP4 diffusion porometer (Delta-TDevices Ltd., Cambridge, UK). All measurements were performed on leaves of 6 plants per treatment between 10 a.m. and 2 p.m.

### Malondialdehyde, proline, and total soluble sugars quantification

Shoot malondialdehyde (MDA) was quantified by the thiobarbituric acid reaction as reported in Heath and Packer [41]. For each sample, *c*.*a*. 250 mg of FW were ground in liquid nitrogen. A solution of 1.25% glycerol and 5% thicloroacetic acid was added to the powder. Samples were then centrifuged at 4 °C during 10 min at 6,700 *g*. Solutions were filtered with one layer of Whatman paper (Grade 1) and 2 mL of the filtrate were mixed with 2 mL of 0.67% thiobarbituric acid. Samples were incubated at 100 °C during 30 min and then cooled on ice. Absorbance was read at 532 nm using a Beckman DU640 spectrophotometer and values related to non-specific absorbance (600 nm) were subtracted. A molar extinction coefficient of 155 mM^-1^ cm^-1^ was used to calculate MDA concentrations.

Shoot proline was quantified using the ninhydrin method according to Bates et al. [42]. For each sample, *c*.*a*. 200 mg FW of shoots were ground in liquid nitrogen in a mortar containing 10 mL of 3% sulfosalicylic acid. Samples were then centrifuged at 1,000 *g* for 5 min and 2 mL of the supernatant were incubated at 100 °C in the presence of 2 mL ninhydrin and 2 mL acetic acid. After extraction with toluene (2 mL), proline was quantified at 520 nm with a Beckman DU640 spectrophotometer using proline standards (SigmaAldrich).

Total soluble sugars were quantified in shoot using anthrone method as previously described by Yemm and Willis [43]: *c*.*a*. 300 mg FW of leaves were ground in liquid nitrogen. The obtained powder was mixed with 7 mL of ethanol 70% (w/v) for 5 min on ice and centrifuged at 4 °C at 8,000 *g* during 20 min. Then, 200 μL of the supernatant reacted with 1 mL of anthrone solution (0.5 anthrone, 250 mL 95% H_2_SO_4_ and 12.5 mL distilled water). A calibration curve was established using glucose as standard and the absorbance was read at 625 nm.

### RNA extraction, reverse transcription-PCR (RT-PCR), and gene expression analysis

At harvest time, shoots and roots of both cultivars were separated and quickly frozen in liquid nitrogen (3 plants were pooled per organ, cultivar and treatment). Total RNA was isolated from 100 mg of frozen material per sample using Trizol reagent protocol (Roche, Germany). The RNA quality and concentration were estimated using NanoDrop ND-1000 (Isogen Life Science, De Meern, The Netherlands). DNase treatments were performed using RQ1 RNase-free DNase (Promega, Leiden, The Netherlands) according to the manufacturer’s instructions. Reverse transcription was performed with 1 μg of total RNA using the GoScriptTM Reverse Transcription Mix, Oligo (dT) Protocol Kit (Promega Benelux b.v., Leiden, The Netherlands) following the manufacturer’s instructions.

Then, six independent PCR amplifications were conducted for each gene using primer pairs, annealing temperatures, and number of cycles as presented in Table 1. The Na^+^ transporter genes were selected from the list established by Irakoze [44] while chloride transporter genes were identified based on the work of Nakamura et al. [45] and Teakle and Tyerman [17].

**Table 1:** List of primers corresponding to *OsHKT1*.*5*, *OsHKT1*.*1*, *OsSOS1*, *OsSOS2*, *OsNHX1*, *OsNHX2*, *OsNRT1*, *OsCLC*, *OsCCC1*, *OsDREB2*, *OsEF1*α and amplification conditions used for semi-quantitative RT-PCR expression analysis.

Gene	NCBI accession	Primer	sequence	Tm (°C)	Size (bp)	number of cycle
*OsHKT1*.*5*	JQ695816.1	Forward	5’-CCTGCCACCTTACACCACTT-3’	57	198	38
		Reverse	5’-GCTGTAGTTGATGGGGTCGT-3’			
*OsHKT1*.*1*	AJ491816	Forward	5’-GGAAGTCAATTTCTTCTGATCCA-3’	58	239	39
		Reverse	5’-TCATTTCAGGATGAACTCCTTG-3’			
*OsSOS1*	AY785147	Forward	5’-CTCCGTGCTCATAGAATCGC-3’	58	207	38
		Reverse	5’-ATACTCACTCAAGTGGGTCAATACC-3’			
*OsSOS2*	DQ248963.1	Forward	5′-ATGAAGGTGCTCGACAAGGA-3’	58	168	38
		Reverse	5′-GCCTCCAGTGATAAGCTCCA-3′			
*OsNHX1*	AB021878	Forward	5’-GTTCAAGAGTTACAACAAAGCACG-3’	58	200	38
		Reverse	5’-CAGCGGGAATACAAAAGCAG-3’			
*OsNHX2*	AY360145	Forward	5’-ACCAAGACGAAACACCCCTAC-3’	57	196	38
		Reverse	5’-AACCCAGCAACTACTCCAAGAA-3’			
*OsNRT1*	AF140606.1	Forward	5’-TGGAGAAACACATCGGCTCT-3’	58	213	37
		Reverse	5’-CCACCAATGCTGCTGATACC-3’			
*OsCLC*	DQ272692.1	Forward	5’-AGTTCGTCGTCACCTCCAAT-3’	57	174	36
		Reverse	5’-CAACCCCGTTCAAGTAAGCC-3’			
*OsCCC1*	LC085614.1	Forward	5’-TCTATTTGGAGCCTTGGCCA-3’	57	162	37
		Reverse	5’-GCTGCCAGTAATCTTGGAGC-3’			
*OsDREB2*	AF300971.2	Forward	5’-GGTGGAAGGAGCAAAACCAG-3’	57	181	38
		Reverse	5’-CGGTTTGGTTCACGGATCTC-3’			
*OsEF1α*	D63580.1	Forward	5’-TCCTCAAGAACGGTGATGCT-3’	56	164	33
		Reverse	5’-GGTTGGGTCCTTCTTCTCCA-3’			

Primers of each studied gene were designed using Primer 3 software (Table 1) [46]. Amplifications were conducted using GoTaq DNA polymerase (Promega Benelux b.v., Leiden, The Netherlands). The PCR products were resolved on agarose gels and expression differences were analyzed by gel densitometry using ImageJ software and expressed as relative values compared to *OsEF1α* expression (peak size of target gene/peak size of *OsEF1α;*) [47]. For a given sample, gene expression analyses were repeated six times and provided similar results.

### Data analysis

The experiment was repeated three times and led to statistical similar results. All the data in this research were analyzed with JMP Pro 14 software. Normality distributions and homoscedasticity were verified using Shapiro-Wilk and Levene’s tests respectively and data were transformed when required. Data were analyzed using two-way or three-way analysis of variance (ANOVA). When significant differences were observed (*P* < 0.05), post-hoc Tukey’s HSD test was performed using all-pairwise comparisons to compare means. Principal Component Analysis (PCA) was performed with the software R version 4.2.1 with the package Factoshiny.

## Results

### Plant growth, water status and photosynthetic parameters

Treatments had no impact neither on the relative increase in the number of leaves nor on the root WC (Table 2). Salinity (NaCl 75 mM) reduced the shoot elongation in both cultivars, while the MIX treatment (NaCl + PEG) reduced it in TOG5949 only, as shown in Table 2 for 7 days of treatment.

**Table 2:** Impact of iso-osmotic solutions of PEG 12.9%, NaCl 75 mM or MIX treatment (PEG 6.4% + NaCl 37.5 mM) applied for 7 days on relative increase in the number of leaves, relative elongation of shoots and roots, shoot and root dry weights, shoot and root water content of two contrasting cultivars of African rice (*Oryza glaberrima* Steud. cv TOG5307 (salt-tolerant) and TOG5949 (salt-sensitive)). Each value is the mean of 6 replicates ± SE. For a given cultivar, values followed by similar letter are not statistically different according to Tukey post hoc test.

Cultivars	Treatment	Duration	Relative increase in number of leaves	Relative elongation		Dry weight (g)		Water content (%)	
				Shoot	Root	Shoot	Root	Shoot	Root
TOG5307	Control	7 days	0.40 ± 0.06 a	0.88 ± 0.05 a	1.18 ± 0.04 a	0.05 ± 0.003 a	0.02 ± 0.001 a	89.07 ± 0.94 a	81.32 ± 1.57 a
TOG5949			0.17 ± 0.07 a	0.91 ± 0.01 a	1.09 ± 0.07 a	0.05 ± 0.002 a	0.01 ± 0.001 a	84.86 ± 0.82 a	75.29 ± 6.52 a
TOG5307	PEG		0.33 ± 0.07 a	0.89 ± 0.04 a	0.64 ± 0.09 b	0.04 ± 0.002 b	0.02 ± 0.001 a	90.14 ± 0.52 a	75.32 ± 0.68 a
TOG5949			0.17 ± 0.07 a	0.86 ± 0.04 a	0.34 ± 0.09 b	0.03 ± 0.003 b	0.01 ± 0.001 a	87.24 ± 1.25 a	73.74 ± 1.55 a
TOG5307	NaCl		0.27 ± 0.03 a	0.31 ± 0.04 b	1.22 ± 0.06 a	0.03 ± 0.001 b	0.01 ± 0.001 b	75.82 ± 1.81 b	87.75 ± 8.99 a
TOG5949			0.13 ± 0.03 a	0.18 ± 0.03 b	0.40 ± 0.09 b	0.02 ± 0.002 c	0.01 ± 0.001 b	83.64 ± 0.92 a	84.65 ± 7.94 a
TOG5307	MIX		0.33 ± 0.07 a	0.75 ± 0.04 a	0.90 ± 0.1 ab	0.03 ± 0.003 b	0.01 ± 0.001 b	85 ± 1.26 a	85.28 ± 1.39 a
TOG5949			0.13 ± 0.03 a	0.27 ± 0.05 b	0.65 ± 0.05 b	0.02 ± 0.002 c	0.01 ± 0.001 b	85.72 ± 0.97 a	83.25 ± 2.37 a

PEG reduced root elongation in both cultivars and both NaCl and MIX treatments affected root elongation in TOG5949 but not in TOG5307. All treatments had a deleterious impact on the shoot DW while only NaCl and MIX treatments reduced the root DW. S1 Table indicated that a significant interaction between cultivars and treatments was recorded for leaf and root elongations, as well as root FW, confirming the higher sensitivity of TOG5949 comparatively to TOG5307. In contrast the shoot WC was unexpectedly reduced by NaCl treatment in TOG5307 only.

The maximal efficiency of PSII photochemistry in the dark-adapted state (*F*_v_/*F*_m_) (Table 3), the actual PSII efficiency (Ф_PSII_) and the photochemical quenching coefficient (qP) were not affected by stresses after one day of treatment but the non-photochemical quenching (NPQ) increased in response to PEG to a higher extent in TOG5949 than in TOG5307. After 7 days of treatment, PEG reduced *F*_v_/*F*_m_, Ф_PSII_, and qP in TOG5949, but not in TOG5307. NaCl and MIX treatments reduced these parameters in both cultivars but the recorded decreases were significantly higher in TOG5949 than in TOG5307 (Table 3, S1 Table). Similarly, PEG increased NPQ in TOG5949 while NaCl and MIX treatments increased it in both cultivars.

**Table 3:** Impact of iso-osmotic solutions of PEG 12.9%, NaCl 75 mM or MIX treatment (PEG 6.4% + NaCl 37.5 mM) applied for 1 or 7 days on maximal efficiency of PSII photochemistry in the dark-adapted state (*F*_v_/*F*_m_), actual PSII efficiency (Ф_PSII_), photochemical quenching (q_P_) and non-photochemical quenching (NPQ) of two contrasting cultivars of African rice (*Oryza glaberrima* Steud. cv TOG5307 (salt-tolerant) and TOG5949 (salt-sensitive)). Each value is the mean of 6 replicates ± SE. For a particular cultivar, values followed by similar lower case (1 day) or uppercase (7 days) letters are not statistically different according to Tukey post hoc test.

Cultivars	Treatment	Duration	*F*_v_/*F*_m_	Ф_PSII_	qP	NPQ
TOG5307	Control	1 day	0.87 ± 0.004 a	0.78 ± 0.007 a	0.92 ± 0.014 a	0.25 ± 0.008 b
TOG5949			0.85 ± 0.008 a	0.78 ± 0.006 a	0.92 ± 0.009 a	0.25 ± 0.007 b
TOG5307	PEG		0.86 ± 0.002 a	0.82 ± 0.010 a	0.96 ± 0.009 a	0.28 ± 0.008 a
TOG5949			0.84 ± 0.004 a	0.79 ± 0.011 a	0.93 ± 0.015 a	0.34 ± 0.007 a
TOG5307	NaCl		0.87 ± 0.005 a	0.81 ± 0.005 a	0.92 ± 0.010 a	0.21 ± 0.009 bc
TOG5949			0.87 ± 0.006 a	0.78 ± 0.004 a	0.92 ± 0.006 a	0.24 ± 0.005 b
TOG5307	MIX		0.87 ± 0.006 a	0.81 ± 0.003 a	0.93 ± 0.005 a	0.20 ± 0.004 c
TOG5949			0.87 ± 0.005 a	0.81 ± 0.004 a	0.92 ± 0.013 a	0.26 ± 0.010 b
TOG5307	Control	7 days	0.86 ± 0.003 A	0.78 ± 0.009 A	0.92 ± 0.006 A	0.21 ± 0.012 C
TOG5949			0.86 ± 0.005 A	0.78 ± 0.008 A	0.92 ± 0.003 A	0.21 ± 0.010 D
TOG5307	PEG		0.84 ± 0.005 A	0.76 ± 0.011 AB	0.89 ± 0.006 A	0.25 ± 0.008 C
TOG5949			0.81 ± 0.004 B	0.73 ± 0.015 B	0.88 ± 0.006 B	0.32 ± 0.007 C
TOG5307	NaCl		0.79 ± 0.002 C	0.71 ± 0.004 B	0.85 ± 0.016 B	0.44 ± 0.009 A
TOG5949			0.77 ± 0.004 C	0.69 ± 0.002 B	0.81 ± 0.003 C	0.53 ± 0.013 A
TOG5307	MIX		0.81 ± 0.003 B	0.75 ± 0.012 AB	0.87 ± 0.004 AB	0.36 ± 0.009 B
TOG5949			0.77 ± 0.002 C	0.73 ± 0.010 B	0.82 ± 0.008 C	0.42 ± 0.013 B

After one day of treatment, net photosynthesis (*A*) (Table 4) increased in TOG5307 exposed to PEG and decreased in both cultivars exposed to NaCl and MIX treatments. In the two considered cultivars, *A* values were the lowest for plants exposed to the MIX treatment while NaCl was the most detrimental treatment for plants exposed for 7 days. *A* values were lower in TOG5949 than in TOG5307. A similar trend was recorded for stomatal conductance (*g*_s_), suggesting that photosynthesis inhibition on both a short (1 day) and a long (7 days) term basis may be, at least partly, related to stomatal limitations. It has however to be mentioned that, as far as control plants are concerned, *A* values were higher after 7 days than after 1 day while an opposite trend was reported for *g*_s_, which indicates that intrinsic water use efficiency (*A*/*g*_s_) increased with the age of the plant for the considered developmental stage. Instantaneous transpiration rate (*E*) was marginally reduced in NaCl and MIX stressed plants after 1 day. In contrast, after 7 days it was dramatically reduced in all stressed plants (Table 4), reaching the minimal value in response to NaCl. In all stressed plants *E* values were significantly higher in TOG5307 than in TOG5949.

**Table 4:** Impact of iso-osmotic solutions of PEG 12.9%, NaCl 75 mM or mix treatment (PEG 6.4% + NaCl 37.5 mM) applied for 1 or 7 days on net photosynthesis (*A*), instantaneous transpiration (*E*), stomatal conductance (*g*_s_), malondialdehyde (MDA), proline, and total sugars concentration of two contrasting cultivars of African rice (*Oryza glaberrima* Steud. cv TOG5307 (salt-tolerant) and TOG5949 (salt-sensitive)). Each value is the mean of 6 replicates ± SE. For a given cultivar, values followed by similar lower case (1 day) or uppercase (7 days) letters are not statistically different according to Tukey post hoc test.

Cultivars	Treatment	Duration	*A* (μmol CO_2_ m^-2^ s^-1^)	*E* (mmol m^-2 s-1^)	*g*_s_ (mmol H_2_O m^-2^ s^-1^)	Shoot *Ψs* (MPa)	Root *Ψ*s (MPa)	MDA (mmol g^-1^ FW)	Proline (μmol g^-1^ FW)	Total sugars (mg g^-1^ FW)
TOG5307	Control	1 day	3.87 ± 0.07 b	3.50 ± 0.10 a	697 ± 11 a	-1.26 ± 0.03 a	-0.65 ± 0.03 a	1.54 ± 0.07 a	1.83 ± 0.08 d	9.43 ± 0.30 ab
TOG5949			3.98 ± 0.12 a	3.43 ± 0.09 a	688 ± 13 a	-1.26 ± 0.03 a	-0.66 ± 0.02 a	1.47 ± 0.09 b	1.95 ± 0.11 c	9.58 ± 0.27 c
TOG5307	PEG		4.93 ± 0.12 a	3.49 ± 0.10 a	580 ± 6 c	-1.30 ± 0.01 a	-0.82 ± 0.02 b	1.64 ± 0.08 a	3.85 ± 0.08 a	9.63 ± 0.16 ab
TOG5949			3.89 ± 0.09 a	3.30 ± 0.11 ab	237 ± 8 c	-1.61 ± 0.03 b	-0.89 ± 0.02 c	1.71 ± 0.05 a	2.64 ± 0.18 b	10.51 ± 0.58 c
TOG5307	NaCl		3.06 ± 0.11 c	3.18 ± 0.09 b	317 ± 5 d	-1.52 ± 0.04 b	-0.81 ± 0.03 b	1.63 ± 0.04 a	3.18 ± 0.06 c	9.89 ± 0.15 a
TOG5949			2.94 ± 0.08 b	2.99 ± 0.09 c	282 ± 6 b	-1.66 ± 0.03 b	-0.90 ± 0.01 c	1.65 ± 0.07 a	3.14 ± 0.08 a	11.32 ± 0.52 a
TOG5949	MIX		2.27 ± 0.08 d	3.27 ± 0.14 ab	255 ± 16 b	-1.29 ± 0.04 a	-0.78 ± 0.01 b	1.70 ± 0.08 a	3.02 ± 0.10 b	10.23 ± 0.37 b
TOG5307			2.46 ± 0.12 c	3.04 ± 0.08 b	238 ± 9 c	-1.56 ± 0.03 b	-0.79 ± 0.03 b	1.58 ± 0.07 a	3.52 ± 0.08 a	8.96 ± 0.27 bc
TOG5307	Control	7 days	5.59 ± 0.16 A	3.35 ± 0.08 A	389 ± 13 A	-1.55 ± 0.01 A	-0.71 ± 0.01 A	3.12 ± 0.26 C	2.93 ± 0.10 D	9.79 ± 0.35 B
TOG5949			5.55 ± 0.09 A	3.36 ± 0.12 A	383 ± 8 A	-1.58 ± 0.02 A	-0.70 ± 0.01 A	3.25 ± 0.18 C	3.01 ± 0.24 D	10.10 ± 0.91 B
TOG5307	PEG		3.49 ± 0.09 B	1.97 ± 0.12 B	291 ± 8 B	-1.91 ± 0.09 B	-0.81 ± 0.02 B	5.49 ± 0.41 B	8.46 ± 0.32 C	14.18 ± 0.56 A
TOG5949			3.15 ± 0.05 B	1.70 ± 0.10 B	225 ± 9 B	-2.42 ± 0.05 B	-0.84 ± 0.01 B	11.97 ± 0.4 B	6.42 ± 0.59 C	15.85 ± 0.8 A
TOG5307	NaCl		1.82 ± 0.13 C	0.90 ± 0.09 B	154 ± 6 D	-2.88 ± 0.03 D	-0.87 ± 0.01 C	15.8 ± 0.76 A	21.29 ± 1.12 A	14.23 ± 0.64 A
TOG5949			0.90 ± 0.11 D	0.67 ± 0.03 D	123 ± 6 C	-3.42 ± 0.06 D	-0.88 ± 0.01 B	19.2 ± 0.88 A	16.56 ± 1.04 A	16.23 ± 0.64 A
TOG5307	MIX		3.29 ± 0.10 B	1.80 ± 0.10 C	253 ± 8 C	-2.22 ± 0.09 C	-0.8 ± 0.01 BC	4.8 ± 0.7 B	13.96 ± 0.74 B	13.69 ± 0.68 A
TOG5949			2.22 ± 0.08 C	1.26 ± 0.03 C	237 ± 8 B	-2.79 ± 0.10 C	-0.85 ± 0.01 B	10.93 ± 0.72 B	11.34 ± 0.66 B	15.89 ± 0.61 A

The shoot *Ψ*s (Table 4) decreased after 1 day of exposure to PEG and MIX treatments in TOG5949 and decreased in both cultivars in response to NaCl treatments, the lowest value being observed for TOG5949 exposed to NaCl. All treatments decreased shoot *Ψ*s after 7 days of exposure, and the values recorded for TOG5949 were always lower than those observed for TOG5307. The root *Ψ*s also decreased in stressed plants of both cultivars, but no significant interaction between cultivars and treatment, or between cultivars and stress duration was recorded for this parameter (S1 Table).

### Mineral concentrations

**Fig 1:**
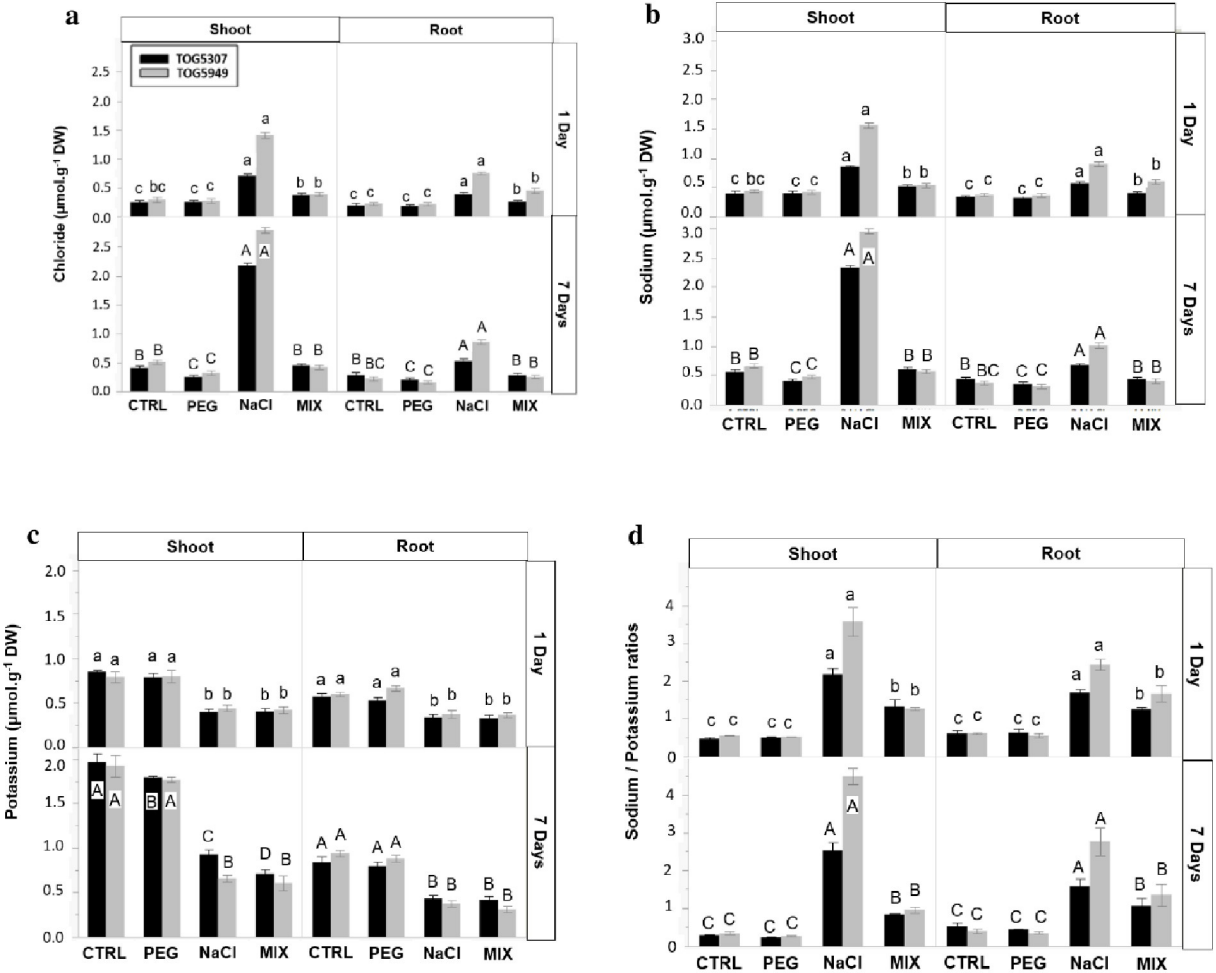
**(a)** Chloride, **(b)** Sodium, **(c)** Potassium concentrations, and **(d)** Na:K ratio in roots and shoots of African rice seedlings (*Oryza glaberrima* Steud.) from cv. TOG5307 (black bars) and TOG5949 (grey bars) cultivated during 1 and 7 days in control conditions (CTRL) or in the presence of 75 mM NaCl, 12.9% PEG-6000 or MIX (6.4% PEG + 37.5 mM NaCl). Each value is the mean of three replicates per treatment and vertical bars are standard errors of the mean. Treatments followed by the same lowercase latter (1 day) or the same uppercase (7 days) for a particular cultivar and a given organ do not differ statistically according to the Tukey post hoc test.

Chloride concentrations (Fig 1a) increased in response to NaCl and MIX treatment for both roots and shoots. TOG5949 exhibited a higher Cl^-^ concentration than TOG5307 under NaCl stress. Moreover, it has to be noticed that Cl^-^ accumulation was already significant after 1 day of stress exposure and that Cl^-^ accumulation recorded for the MIX treatment remained quite limited comparatively to NaCl exposure. After 7 days of exposure, root and shoot Cl^-^ concentrations recorded for the MIX treatment were indeed similar to control plants for both cultivars.

The NaCl treatment also induced a strong increase in shoot and root Na^+^ concentrations (Fig 1b). Throughout the experiment, TOG5307 accumulated less sodium than TOG5949 in both organs. Once again, Na^+^ accumulation after 7 days of treatment was quite low in response to MIX treatment and was even not significant after 7 days of treatment. The NaCl and MIX treatments induced an obvious decrease in K^+^ concentration which was recorded already after 1 day of exposure (Fig 1c). After 1 day, the recorded decrease was similar in response to NaCl and MIX treatments for both roots and shoots and for the two tested cultivars. A similar trend was recorded after 7 days, except that the K^+^ concentration significantly decreased in response to PEG in TOG5307 and that the K^+^ decrease recorded at the shoot level for this cultivar was higher for MIX than for NaCl treatment. As an overall consequence, the Na^+^/K^+^ ratio (Fig 1d) was not affected by PEG comparatively to control and was similar in the two tested cultivars. In response to NaCl treatment, Na^+^/K^+^ ratio was clearly higher in TOG5949 than in TOG5307. Na^+^/K^+^ ratio in response to MIX treatment was intermediate between NaCl and control.

### Malondialdehyde, proline, and total soluble sugars concentrations

The shoot MDA concentration remained unaffected after 1 day of treatment but it drastically increased in stressed plants after 7 days (Table 4). NaCl-stressed plants accumulated the highest concentrations of MDA, with a maximal value recorded in TOG5949. The shoot MDA concentrations of plants exposed to PEG and MIX treatments were almost double in TOG5949 comparatively to TOG5307.

The shoot proline concentration slightly increased in all stressed plants (Table 4) after 1 day of treatment. Although being significant in all cases, such an increase remained extremely limited from a relative point of view. The maximal shoot proline concentration was recorded in TOG5307 exposed to PEG. In contrast, shoot proline significantly accumulated after 7 days of stress exposure, with the highest accumulation in NaCl-treated plants. The MIX treatment presented intermediate value while the PEG-treated plants accumulated the lowest amount of proline among stressed plants. In all cases, shoot proline concentration was higher in TOG5307 than in TOG5949.

After 1 day of treatment, total soluble sugar remained unaffected in TOG5307 and slightly increased in NaCl-treated plants of TOG5949, only. Accumulation was more marked after 7 days and appeared similar in all stressed plants, with a higher concentration in TOG5949 than in TOG5307 (Table 4).

### Relative expression of genes: Semi-quantitative RT-PCR expression analysis

**Fig 2:**
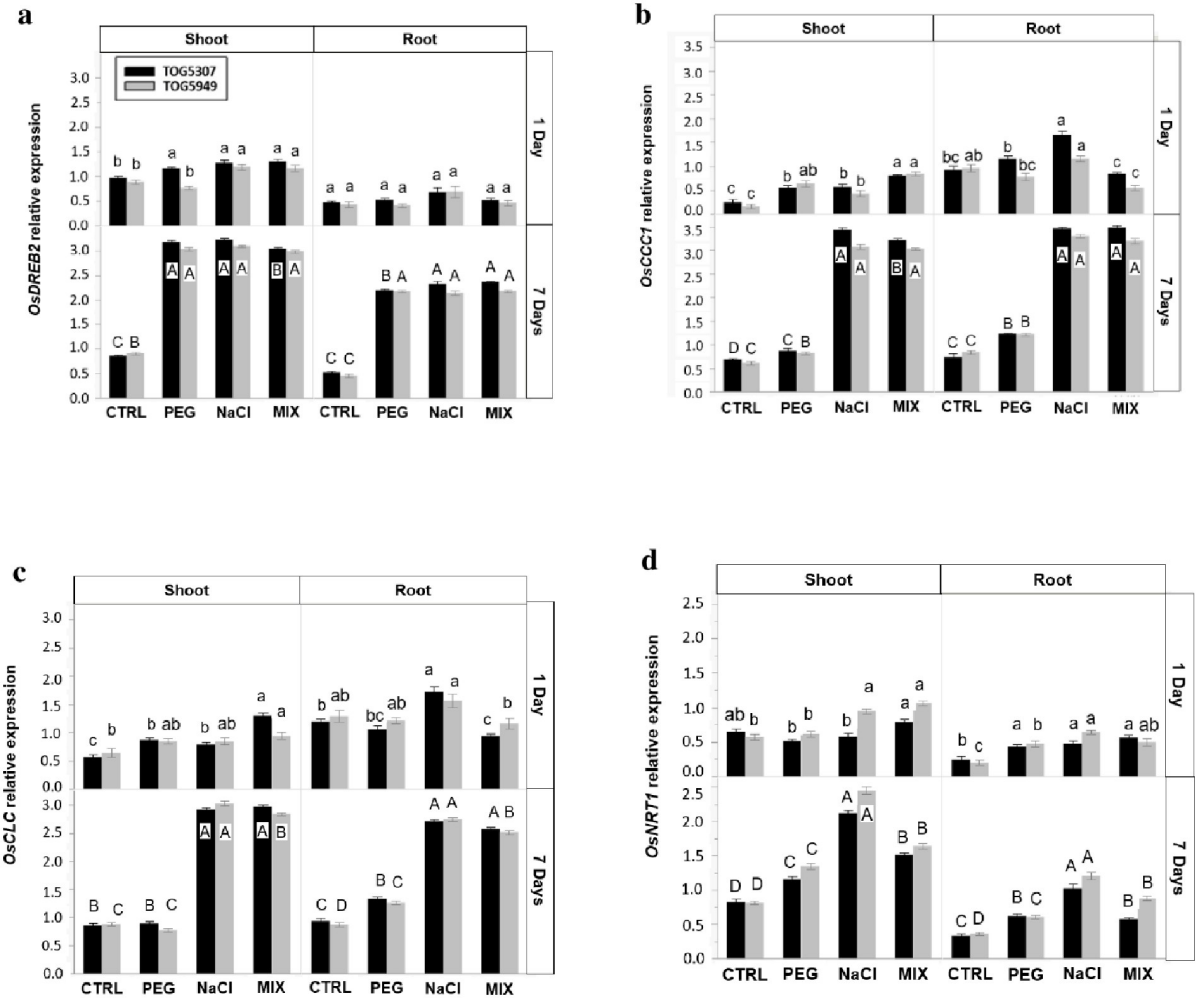
Semi-quantitative RT-PCR expression analysis of **(a)** *OsDREB2*, **(b)** *OsCCC1*, **(c)** *OsCLC*, and **(d)** *OsNRT1* genes in roots and shoots of African rice seedlings (*Oryza glaberrima* Steud.) from cv. TOG5307 (black bars) and TOG5949 (grey bars) cultivated during 1 and 7 days in control conditions (CTRL) or in the presence of 75 mM NaCl, 12.9% PEG-6000 or MIX (6.4% PEG +37.5 mM NaCl). Each value is the mean of three replicates per treatment and vertical bars are standard errors of the mean. Treatments followed by the same lowercase latter (1 day) or the same uppercase (7 days) for a particular cultivar and a given organ do not differ statistically according to the Tukey post hoc test.

*OsDREB2* was only slightly induced in the shoots after 1 day of exposure but it was highly induced in both shoot and roots after 7 days (Fig 2a). At this time, the expression of *OsDREB2* was similar for all considered stress treatments in both organs. Nevertheless, cultivars had a significant impact of *OsDREB2* expression (S1 Table) which was indeed slightly higher in TOG5307 comparatively to TOG5949.

After 1 day exposure, *OsCCC1* coding for a cation-chloride-cotransporter was significantly induced by NaCl treatment in the roots of TOG5307 (Fig 2b) while the NaCl-induced increase in TOG5949 was not significant. This gene was repressed in the roots of TOG5949 exposed to the MIX treatment. At the shoot level, this gene expression was slightly increased, the highest value being recorded for the MIX treatment. A very high expression of *OsCCC1* was recorded after 7 days of exposure to NaCl and MIX treatments in both roots and shoots of the two considered cultivars. It is noteworthy that PEG treatment also slightly increased the gene expression. No significant difference was recorded between cultivars for this specific gene expression (S1 Table).

*OsCLC* is coding for another Cl^-^ transporter (Fig 2c). It was induced after 1 day of stress in the roots of NaCl-treated TOG5307 but repressed in the MIX treatment in this cultivar. In contrast, as far as shoot are concerned, the highest induction was recorded for plants exposed to the MIX treatment in both cultivars. After 7 days of treatment, a very high induction of *OsCLC* expression was noticed in roots and shoots of the two cultivars exposed to NaCl or to the MIX treatments. *OsCLC* was also induced by PEG treatment in the roots but not in the shoots.

*OsNRT1* was induced to a higher extent in the shoots than in the roots (Fig 2d). After 1 day of treatment, the recorded increase in the shoots was significant for TOG5949 exposed to NaCl and MIX treatments. After 7 days of treatment, *OsNRT1* expression increased in response to all stressing agents. The highest values were recorded for NaCl-treated plants and the lowest stimulation was observed in PEG-exposed ones, MIX treatment exhibiting intermediate values. Cultivars had a significant impact on this parameter (S1 Table) and the gene expression in response to NaCl and MIX treatments was higher in TOG5949 than in TOG5307.

**Fig 3:**
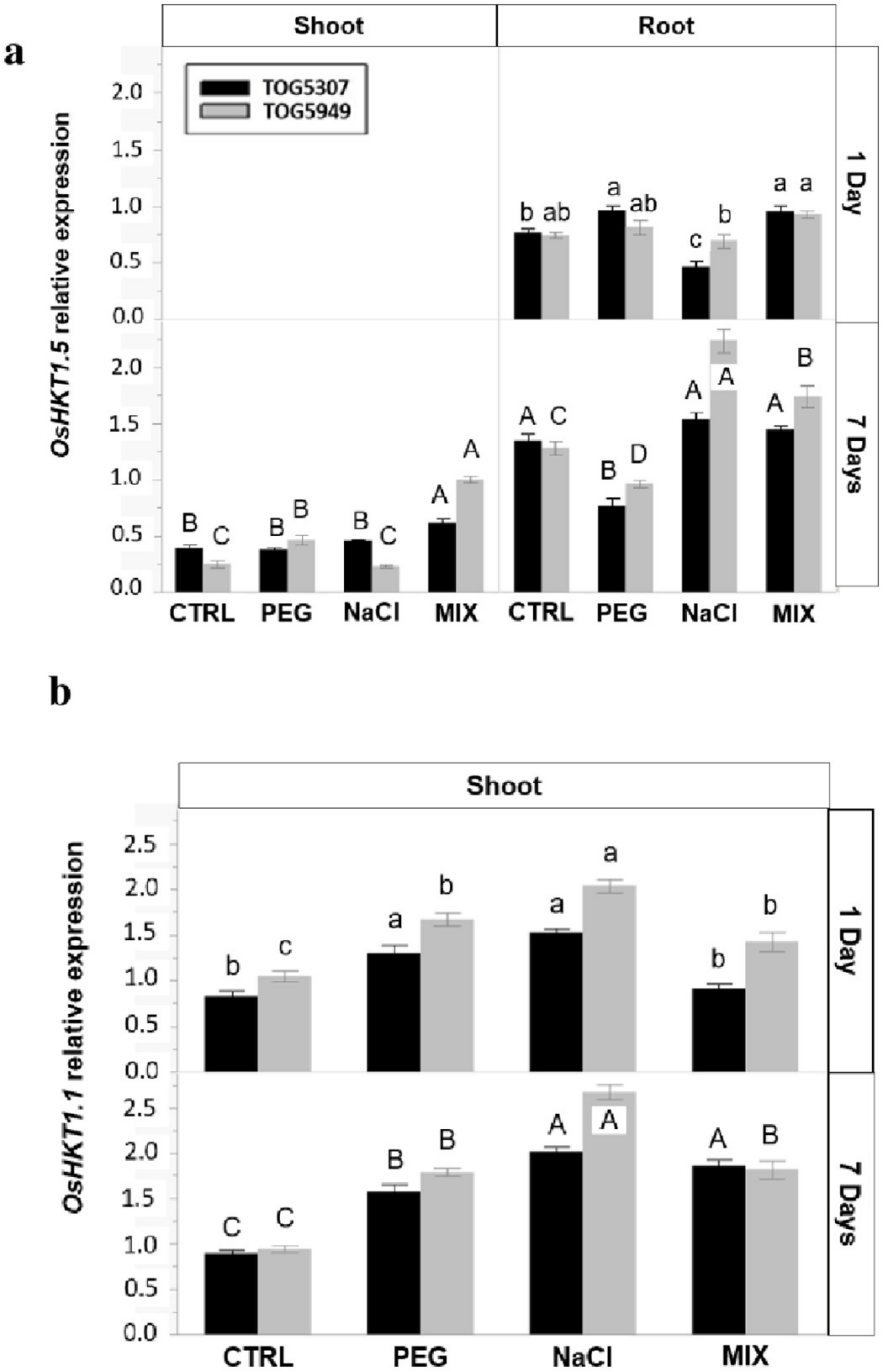
Semi-quantitative RT-PCR expression analysis of **(a)** *OsHKT1*.5, and **(b)** *OsHKT1*.*1*. genes in African rice seedlings (*Oryza glaberrima* Steud.) from cv. TOG5307 (black bars) and TOG5949 (grey bars) cultivated during 1 and 7 days in control conditions (CTRL) or in the presence of 75 mM NaCl, 12.9% PEG-6000 or MIX (6.4% PEG +37.5 mM NaCl). Each value is the mean of three replicates per treatment and vertical bars are standard errors of the mean. Treatments followed by the same lowercase latter (1 day) or the same uppercase (7 days) for a particular cultivar and a given organ do not differ statistically according to the Tukey post hoc test.

*OsHKT1*.*5* expression (Fig 3a) was not detected in the shoots after 1 day of exposure. At this time, the gene expression was significantly increased in the roots of TOG5307 exposed to PEG and MIX treatments but was repressed in the roots of TOG5307 exposed to NaCl. In contrast, stressing treatments had no significant impact on the gene expression at the root level of TOG5949. After 7 days of treatment, the gene expression was detected at the shoot level (including in controls) but remained unaffected in the shoots of TOG5307 exposed to NaCl and PEG treatment while it significantly increased in the shoots of both cultivars exposed to the MIX treatment. At the root level, this gene expression was reduced in response to PEG in both cultivars. The NaCl and MIX treatments significantly increased the gene expression in the roots in TOG5949.

The expression of *OsHKT1*.*1* was detected in the shoots but remained undetectable in the roots (Fig 3b). After 1 day of treatment, the expression increased in the salt-sensitive cultivar TOG5949 in response to all stress treatments, the highest expression being recorded in NaCl-treated plants. In the salt-tolerant TOG5307, it was increased in response to PEG and NaCl stress, but not in response to MIX treatment. After 7 days of exposure, the expression of *OsHKT1*.*1* was increased in all cultivars and in response to all treatments. The expression was still the highest in NaCl-treated plants and was higher in TOG5949 than in TOG5307.

**Fig 4:**
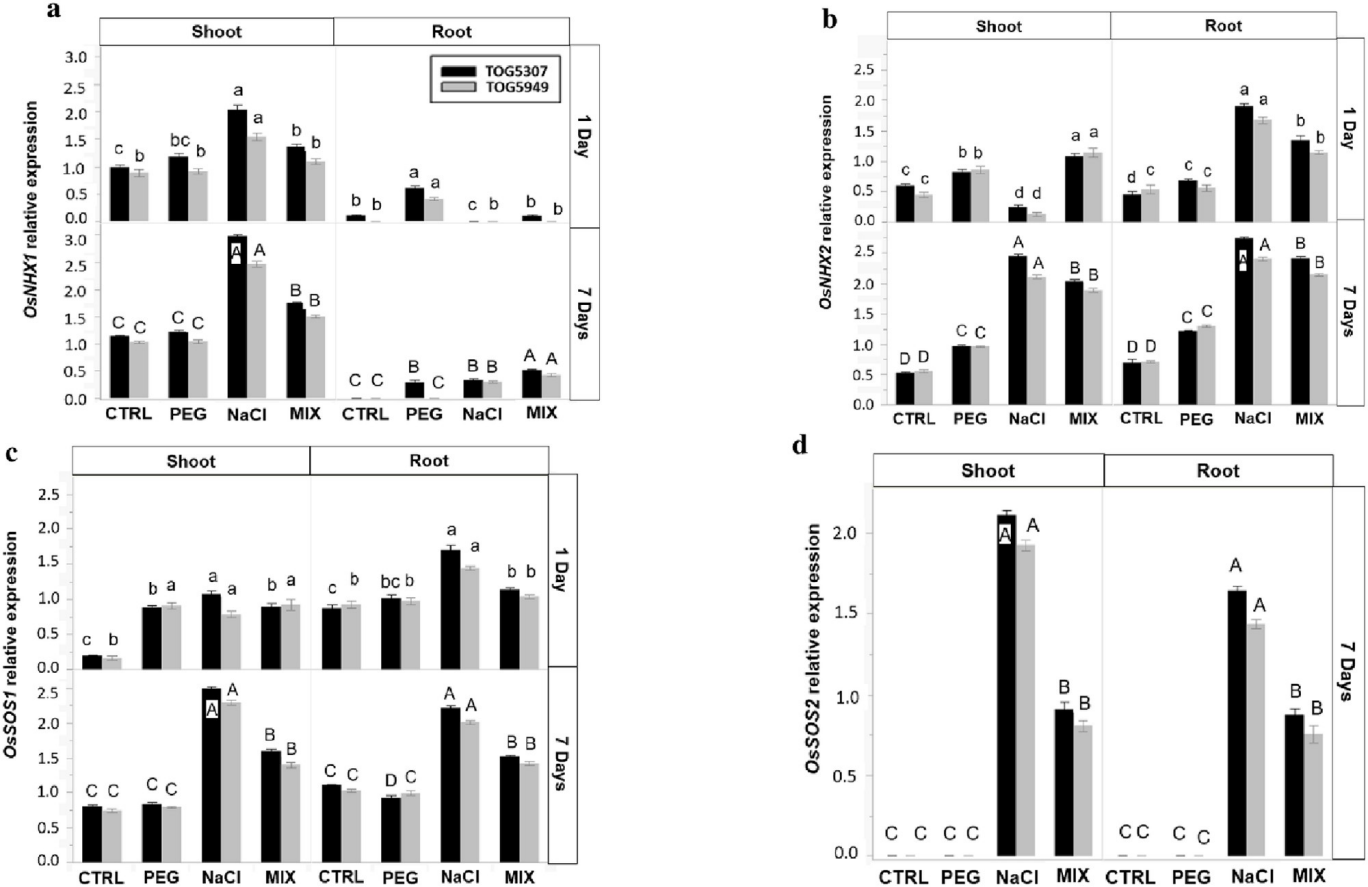
Semi-quantitative RT-PCR expression analysis of **(a)** *OsNHX1*, **(b)** *OsNHX2*, **(c)** *OsSOS1*, and **(d)** *OsSOS2* genes in shoots and roots of African rice seedlings (*Oryza glaberrima* Steud.) from cv. TOG5307 (black bars) and TOG5949 (grey bars) cultivated during 1 and 7 days in control conditions (CTRL) or in the presence of 75 mM NaCl, 12.9% PEG-6000 or MIX (6.4% PEG +37.5 mM NaCl). Each value is the mean of three replicates per treatment and vertical bars are standard errors of the mean. Treatments followed by the same lowercase latter (1 day) or the same uppercase (7 days) for a particular cultivar and a given organ do not differ statistically according to the Tukey post-hoc test.

*OsNHX1* was obviously more expressed in the shoots than in the roots (Fig 4a). After both 1 and 7 days of treatment, the highest expression was observed in the shoots of NaCl-treated plants and was higher in TOG5307 than in TOG5949. PEG treatment had no impact on *OsNHX1* expression, while the MIX treatment increased *OsNHX1* expression in the shoot after 7 days of stress. In the roots of plants treated during 1 day, *OsNHX1* was induced by PEG stress only, while it was slightly increased in response to all treatments after 7 days of treatment, the highest value being recorded for the MIX treatment. After one day of treatment, *OsNHX2* expression (Fig 4b) was reduced in the shoot of plant exposed to NaCl stress while it was significantly induced in plants exposed to PEG and to MIX treatments. In contrast, it was clearly induced at the root level in response to NaCl and to MIX treatments. After 7 days of treatment, expression of *OsNHX2* exhibited a clear stimulation in the shoots and roots of NaCl- and MIX-treated plants, values recorded for TOG5307 being higher than those recorded for TOG5949. PEG increased *OsNHX2* expression in roots and shoots but to a lower extent than other treatments.

After 1 day of treatment, *OsSOS1* expression increased in response to NaCl in the roots of both cultivars (Fig 4c) while MIX treatment slightly increased the root expression of this gene in TOG5307, only. This gene was however overexpressed in the shoots of both cultivars in response to all stressing agents including PEG, already after 1 day of exposure. After 7 days of treatment, NaCl increased *OsSOS1* expression. *OsSOS1* was also slightly overexpressed in response to the MIX-treatment but it remained unaffected in response to PEG. As far as *OsSOS2* is concerned (Fig 4d), the gene expression could not have been accurately quantified in control and PEG-treated plants after one day of stress and data are thus presented for 7 days of treatment, only. *OsSOS2* was strongly overexpressed in response to NaCl in roots and shoots while MIX treatment had a lower impact. In both cases, the gene expression was higher in TOG5307 than in TOG5949. This gene expression remained low in control and was not induced by PEG.

### Principal component analysis

**Fig 5:**
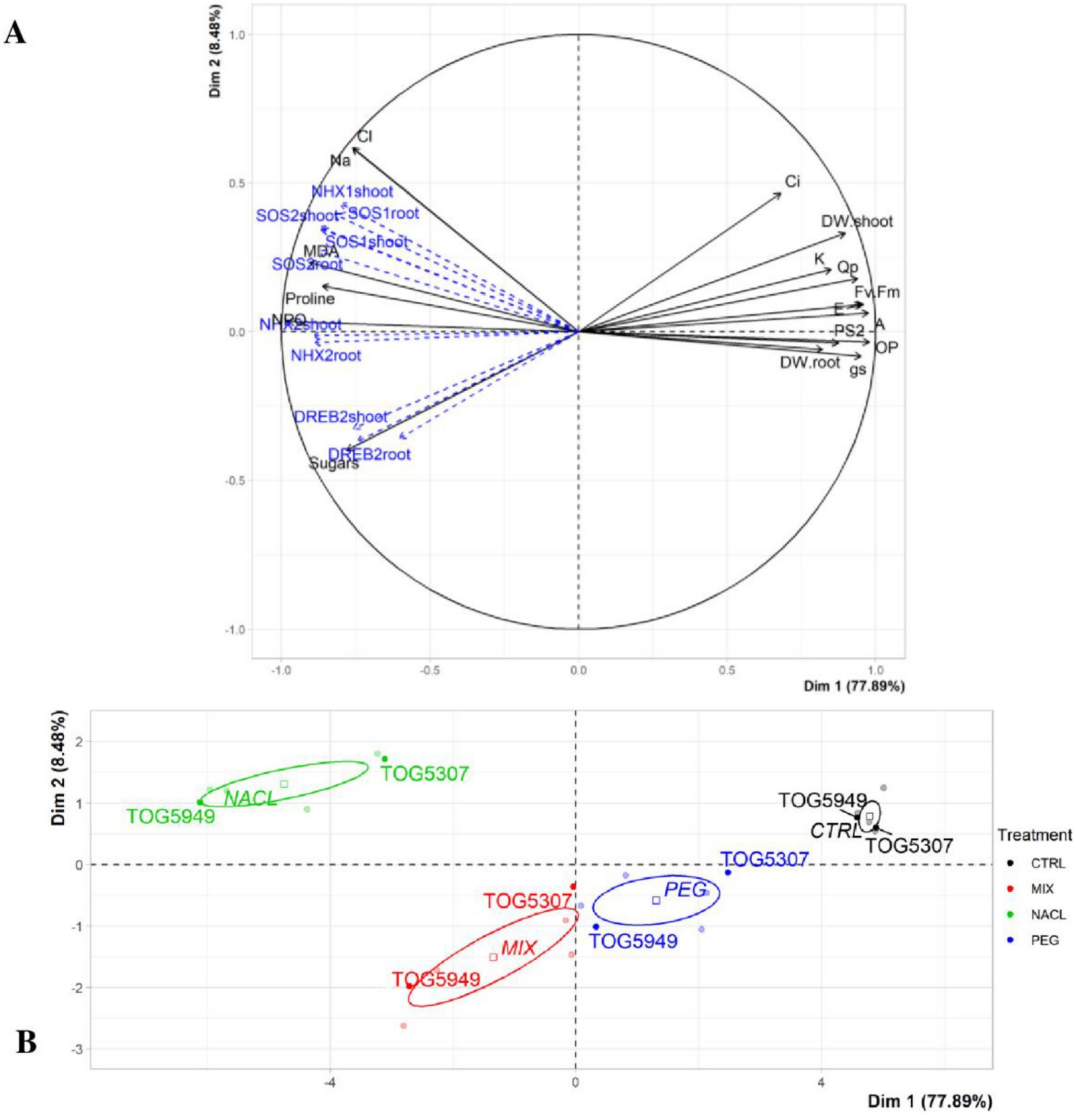
Principal component analysis of agro-physiological and molecular parameters of African rice seedlings (*Oryza glaberrima* Steud.) cv. TOG5307 and TOG5949 cultivated during 7 days in control conditions or in the presence of 75 mM of either NaCl, PEG-6000 or MIX (PEG +NaCl). (A) variable graph: Only significant parameters (*P <* 0.05) with a high quality of representation (cos2 ≥ 0.65) were shown. The molecular data (blue dashed arrow) are here additional variables (they did not contribute to the PCA). (B) Individual graph: Individuals are coloured according to the treatment they have received. Control (CTRL) in black, 75 mM PEG 600 (PEG) in blue, 75 mM NaCl (NACL) in green and PEG + NaCl (MIX) in red. The confidence ellipses drawn around the categories of treatment. Shoot.DW, shoot dry weight; Root.DW, root dry weight; gs, stomatal conductance; K. Potassium concentration; Cl, Chloride concentration; Na, Sodium concentration; Ci, sub-stomatal cavity CO_2_ concentration; E, instantaneous transpiration; A, net photosynthesis; *F*_v_/*F*_m_ maximal efficiency of PSII photochemistry in the dark-adapted state; PS2, actual PSII efficiency; Q_p_, photochemical quenching; NPQ, non-photochemical quenching; OP, osmotic potential; MDA, malondialdehyde concentration; proline, proline concentration; sugars, total sugars concentration.

Principal component analysis (PCA) was performed in order to discriminate the impact of the four treatments on the two African rice varieties. Only agro-physiological parameters collected at 7 days contributed to the design of the PCA. The expressions of the *SOS1*, *SOS2*, *NHX1*, *NHX2* and *DREB2* genes are considered here as additional variables. All displayed variables have a representation quality greater than or equal to 0.65. The PCA revealed that 86.37% of the variance was explained by principal component 1 (Dim 1) and principal component 2 (Dim 2). Principal component 1 alone explained 77.89% of the variance (Fig 5).

Parameters that have the highest value factor coordinate for the Dim 1, with the highest variables contribution, based on correlations, were, at left, toxic ions (Na^+^, Cl^-^), non-photochemical quenching, malondialdehyde, proline and total sugars concentrations. At right, they were sub-stomatal cavity CO_2_ concentration, potassium content, stomatal conductance, instantaneous transpiration, net photosynthesis, maximal efficiency of PSII photochemistry in the dark-adapted state, photochemical quenching, actual PSII efficiency, osmotic potential, root dry weight and shoot dry weight (Fig 5A). The second graph shows the classification of seedlings in response to the treatments in the multivariate PCA space. Dim 1 separates plants that received some NaCl from those that did not: on the left the MIX and NaCl treatments, on the right the CTRL and PEG treatments (Fig 5B). The former (left) showed a positive correlation with toxic ions while the latter (right) showed a positive correlation with argo-morphological and photosynthetic parameters. There was also a positive correlation between the individuals of the MIX and NaCl treatments. Moreover, a positive correlation was between MDA, proline and total sugars on the one hand, and the expression of the *SOS1*, *SOS2*, *NHX1*, *NHX2* and *DREB2* genes on the other hand.

The confidence ellipses drawn on each treatment show that individuals of the TOG5307 cultivar showed a much more positive correlation with the agro-morphological and photosynthetic parameters regardless of the treatment. This was not the case for TOG5949. In the presence of a certain amount of NaCl, individuals of the variety TOG5307 also show a high expression of *SOS1*, *SOS2*, *NHX1*, *NHX2* and *DREB2* genes.

## Discussion

**Fig 6:**
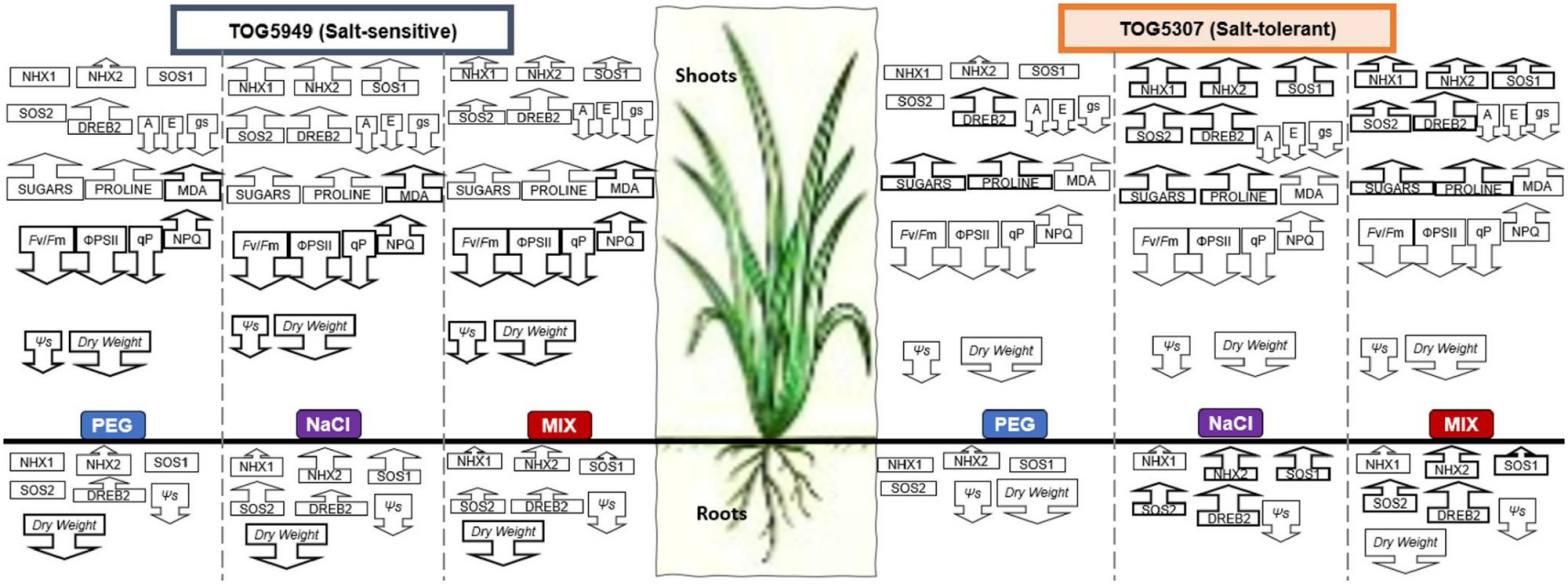
Synthetic overview of the impact of ionic and osmotic toxicity on two African rice cultivars. Plants from the salt-tolerant cultivar TOG5307 and salt-sensitive cultivar TOG5949 were exposed for 7 days to iso-osmotic solution of PEG (12.9%), NaCl (75 mM) or MIX treatment (NaCl 37.5 mM + PEG 6.4%). Direction of the arrow indicates increase or decrease of the corresponding parameter comparatively to non-stressed control of the same cultivar. Bold arrows for a given type of solution indicate discriminating parameters between the two considered cultivars.

Fig 6 presents a synthetic global overview of the differential impact of stressing solution (PEG 12.9%, NaCl 75 mM and MIX (NaCl 37.5 mM + 6.4% PEG) on the behavior of salt-tolerant TOG5307 and salt-sensitive TOG5949.

### *Oryza glaberrima* rapidly respond to the ionic component of salt stress

Plants exposed to environmental constraints may trigger a wide range of strategies to cope with stress. Some of them may be efficient on a short-term basis but are too expensive from an energetical point of view to be maintained for long periods [6]. Plants may then shift from one strategy to another depending on the stress duration [4, 24, 26]. In the present study, stress duration had a significant impact on all recorded parameters except root Na^+^ and Cl^-^ content as well as Na^+^/K^+^ ratios (S1 Table). This suggests that *O*. *glaberrima* is able to rapidly trigger a response to ion toxicities. In numerous studies devoted to salt stress, especially in rice, physiological parameters are recorded after several days or several weeks of treatment, while gene expression is recorded after a very short term exposure (frequently a few hours) to very high and sometimes unrealistic NaCl doses (up to 200 or even 300 mM NaCl) [20, 48, 49]. In most laboratory studies (including this one) stress is imposed according to a one step procedure and plants thus have to cope with an osmotic shock. This is somewhat different of what occurs in field conditions where stress intensity gradually increases, so that only a progressive exposure to increasing NaCl and/or PEG fully reflects field growing conditions [4]. Nevertheless, in the present study, we used a NaCl dose of 75 mM inducing an electrical conductivity comparable to those that may be encountered by rice in salt-affected fields [1, 22–26] and we noticed that the Na^+^/K^+^ ratio reached its final value already after one day of exposure. However, the consequences of ion status on gene expression were quite different after 1 and 7 days, leading us to conclude that stress-induced gene regulation is not only a function of stress intensity but also a function of stress duration and that very short-term transcriptomic approaches are not always fully relevant of plant adaptability.

Taken together, our data provided evidences that TOG53007 was more tolerant to all tested external constraints than TOG5949. As far as salt is concerned, TOG5307 displayed an excluding strategy preventing overaccumulation of toxic Na^+^ and Cl^-^. Some authors nevertheless recently demonstrated that beside such an exclusion strategy, tissue tolerance which implies the maintenance of metabolism despite Na^+^ accumulation may allow to minimize the energy cost of salinity tolerance in rice [27, 49, 50]. It might be argued that exclusion and tolerance are not mutually exclusive and that the most performing plants in salt stress conditions are those which find the best compromise between exclusion process allowing to reduce toxic ion accumulation, and tolerance strategy allowing to cope with accumulated ions.

### Impact of the osmotic component of salt stress on photosynthesis and water status

The use of three types of solutions with similar osmotic potentials (mean *Ψ*s = -0.47 MPa) allowed us to discriminate between the ionic and the osmotic component of stress. TOG5307 is, to some extent, tolerant to partial desiccation: indeed, *A* values remained higher in the leaves of the dehydrated TOG5307 than in TOG5949 (Table 4). The light phase of photosynthesis in terms of chlorophyll-fluorescence related parameters was affected in leaves of TOG5307, but less than in the hydrated leaves of TOG5949, supporting the view of Rane et al. [51] that stability of PSII is an important property related to desiccation tolerance. The instantaneous water use efficiency (*A*/*E*) was 2 μmol CO_2_ mmol^-1^ H_2_O in TOG5307 but only 1.34 μmol CO_2_ mmol^-1^ H_2_O in TOG5949. When exposed to PEG, TOG5307 also behaved better than TOG5949 in terms of net photosynthesis and stomatal conductance, confirming the high tolerance of TOG5307 to water stress. The mechanisms of desiccation tolerance were reported to be triggered by the DREB (Dehydration-Responsive-Element-Binding) proteins [52]. As far as OsDREB2 is concerned, the expression of the corresponding gene was induced to a same extent in response to all treatments after 7 days of stress, suggesting that the parameter triggering overexpression was the external water potential rather than the internal water content. Stress-induced increase in *OsDREB2* expression was also slightly higher in TOG5307 than in TOG5949. According to Chakraborty et al. [53], different members of the DREB family may be differentially induced in response to osmotic constraints and these authors confirmed that DREB2 expression was correlated with drought tolerance in *japonica* rice cultivars. Moreover, Gumi et al. [54] recently identified a gene coding for DREB2A in *O*. *glaberrima* (*OglDREB2A*) and confirmed that it is transcriptionally regulated by both salt and drought stresses.

It is noteworthy that TOG5307 had a higher *Ψ*s value than TOG5949, suggesting that it was less efficient than TOG5949 in terms of osmotic adjustment. Osmotic adjustment may occur through accumulation of soluble compatible organic solutes, although this strategy has a metabolic cost [7, 11]. Proline accumulated to a higher extent in TOG5307 than in TOG5949. In contrast, soluble sugars displayed a higher concentration in TOG5949 than in TOG5307. Proline accumulation only marginally occurred after 1 day of treatment, although *Ψ*s already decreased in the salt-sensitive cultivar TOG5307. Moreover, even after 7 days of exposure, the amount of accumulated proline was too low to explain the measured *Ψ*s decrease. Inorganic ions may also be involved in osmotic adjustment [26, 55]. K^+^ is undoubtedly the major inorganic cation in the cytoplasm where its concentration is in the 100 mM range [2, 11] but it strongly decreased in response to NaCl treatments which hamper osmotic adjustment. Under salt stress conditions, Na^+^ is easily available and may behave as a cheap osmoticum, provided that it is correctly sequestered in vacuoles to prevent its toxicity on cytosolic enzymes [26]. The highest Na^+^ (and Cl^-^) accumulation in salt-sensitive TOG5949 might explain the lowest *Ψ*s value recorded in this cultivar. However, it does not explain why *Ψ*s value was still lower for TOG5949 than for TOG5307 in PEG-treated where no additional Na^+^ is available in the solution.

### Sodium transporters: Absorption, translocation and sequestration

The ion transporters and channels play a key role not only for ion absorption, but also for long-distance translocation, distribution between organs and compartmentation within the cells [9, 10]. Electrochemical gradient at the root surface favors the passive entry of Na^+^, mainly through non-selective cation channels (NSCC). SOS1 ensures a first level of protection since it functions as a Na^+^ excluder, especially at the root tip, although the presence of SOS1 is also reported in the aerial part [10, 11]. SOS1 is activated by SOS2, a serine/threonine-protein kinase, which is itself activated by SOS3 [56]. SOS2 may also interact individually or as a SOS-SOS3 complex with vacuolar transporter of the NHX family. NHXs remove Na^+^ from the cytosol by pumping H^+^ into the vacuole. Several genes coding for NHX proteins have been identified in rice: type-I NHXs are vacuolar-located while type-II NHXs are found at endosome, trans-Golgi network and pre-vacuolar compartments [12]. Type-I NHXs, such as OsNHX1 and OsNHX2 assume crucial function in salinity tolerance through vacuolar sequestration of Na^+^ toxic ions, but this implies the establishment of a H^+^- gradient across tonoplast generated by vacuolar H^+^-ATPase (V-H^+^ ATPAse, EC 3.6.1.34) and by vacuolar H^+^ pyrophosphatase (V-H^+^ PPase, EC 3.6.1.1). Similarly, SOS1 (also referred as NHX7) used an H^+^ gradient across the plasma membrane achieved by plasma membrane H^+^-ATPase (PM-H^+^ATPAse; EC 3.6.1.35). HKTs are high-affinity K^+^ transporter belonging to two subfamilies. In monocotyledonous species, both HKT1 and HKT2 proteins exhibited features of a Na^+^ channel when the external Na^+^ concentrations are high. In rice, OsHKT1.1 and OsHKT1.5 were shown to be Na^+^ selective and are thought to act in Na^+^ retrieval from the xylem, thus contributing to reduce Na^+^ accumulation in the shoots [49]. It has to be noticed that SOS1 has also been identified in xylem parenchyma where it could be involved in Na^+^ xylem retrieval [57]. With the noticeable exception of *OsHKT1;5*, expression of genes coding for those transporters were never studied in response to salt or osmotic stress in *O*. *glaberrima* until now. We took advantage of the genetic proximity between the two cultivated species *Oryza sativa* and *Oryza glaberrima*, which share the A genome from *Oryza* genus to gain additional information in this respect.

The sodium transporter OsHKT1;5 has been identified as a major component of salt tolerance in *Oryza sativa* at both vegetative and reproductive stage [58, 59]. However, in *O*. *glaberrima*, we were unable to detect expression of *OsHKT1;5* in the shoot after 1 day of treatment and it was not strongly expressed in the roots at this period in response to NaCl. After 7 days of exposure, gene expression increased in the roots of the NaCl-sensitive cultivar TOG5949 only, and the gene expression was not stimulated in the shoots. Based on these observations, it seems difficult to attribute a major role to this gene in the response to NaCl for the two tested cultivars. This is puzzling, considering that, according to Platten et al. [35], *HKT1;5* could be involved in Na^+^ exclusion in *O*. *glaberrima*. However, according to these authors, different alleles exist for this gene which strongly influence its impact on salt tolerance in African rice. Similarly in *O*. *sativa*, Jayabalan et al. [59] demonstrated that 4 amino acid changes occurred between the highly salt-tolerant cultivar Nona Bokra and the highly salt-sensitive cultivar Koshihikari and that one single V395 in the vicinity of the sodium selective filter residue strongly enhanced Na^+^ transport rates by reducing pore rigidity in this membrane transporter.

In the present study, *OsHKT1*.*1* expression was detected in the shoot only, and increased in response to all stressing agents. Moreover, its expression was higher in TOG5959 than in TOG5307 and might be regarded as an unsuccessful attempt to retrieve Na^+^ from the xylem sap in order to limit Na^+^ accumulation within the shoots through sodium recycling by the phloem sap. The fact that the expression of this gene increased in response to PEG is at first sight surprising since no Na^+^ excess was present in this treatment. Khan et al. [55] recently reported that another member of the HKT family, *OsHKT1*.*4* is constitutively expressed at very low Na^+^ concentration (0.5 mM) and may assume still unidentified physiological roles at such a low Na^+^ concentrations. Similar functions might occur for HKT1.1 in PEG-treated plants in *O*. *glaberrima*. Imran et al. [49] also demonstrated that the selectivity of HKT transporters for Na^+^ and K^+^ largely depends on a few key amino acid residues and that variants may exhibit strong modification in ion transport activities.

NHX1 and NHX2 are involved in the vacuolar sequestration of toxic Na^+^ and thus protect Na^+^-sensitive enzymes located in the cytosol [8, 9]. *OsNHX1* was only slightly expressed in roots but was more clearly overexpressed in shoots, and to a higher extent in the salt-tolerant cultivar TOG5307 than in the salt-sensitive TOG5949. In contrast, *OsNHX2* was strongly induced in the roots of NaCl-treated plants, already after 1 day of treatment, while high expression in the shoots was recorded after 7 days of stress only. In all cases, expression was higher in TOG5307 than in TOG5949, which supports the view that vacuolar sequestration of Na^+^ indeed plays a role in salinity tolerance. This also supports the view of Zeng et al. [12] that different members of this large family may be differently regulated in the various organs of adult rice plant. The fact that *OsNHX1* and *OsNHX2* were differently expressed in roots and shoots and that their expressions were differently modulated by external factors suggest that the corresponding protein assume non-redundant functions in stressed plants of African rice. There was however no significant interaction between “cultivar” and “stress duration” for *NHX1* and *NHX2* gene expression (S1 Table), suggesting that genes were regulated in similar ways during stress exposure in the two considered cultivars, even if their level of expression differed.

*OsSOS1* was induced in NaCl-treated plants in both cultivars after 1 day of treatment: although its expression was higher in salt-tolerant TOG5307 than in salt-sensitive TOG5949, the difference between the two cultivars remained low from a relative point of view. The highest stimulation was observed in the shoots and this corroborates the hypothesis of Prodjinoto et al. [37] that SOS1 in leaves is involved in Na^+^ extrusion to the apoplasm, especially in the salt-tolerant cultivar. It is interesting to notice that *OsSOS1* gene expression was recorded in all plants (although its expression increased only in the presence of NaCl) while the expression of *OsSOS2*, coding for a dikinase required for SOS1 activation, was detected only after 7 days of exposure to NaCl with a very low expression in control and PEG-treated plants. This was not expected since beside its role in salinity resistance, SOS2 is interacting with H_2_O_2_ signaling protein NDPK2 and is thus involved in ROS signaling. It has also been found to interact with CATALASE 2 and 3 in *Oryza sativa* [56]. Our data suggest that these functions are less crucial for *Oryza glaberrima* than for the Asian rice species.

### Chloride transporters

*CCC1* was very rapidly induced at the root level, and to a higher extent in the salt-tolerant genotype. CCCs are secondary active electroneutral transporters which are functioning as Na^+^:K^+^:2Cl^-^ co-transporters. In the studied cultivars TOG5307 and TOG5949, Prodjinoto et al. [37] used bumetamide as an inhibitor of CCC in *O*. *glaberrima* and concluded that it is a major contributor of Cl^-^ transport, although the counterions appeared to be preferentially K^+^ in the salt-tolerant cultivar and Na^+^ in the salt-sensitive one. According to Teakle and Tyermann [17], this transporter is involved in Cl^-^ xylem retrieval. Even in the absence of salt stress, CCC1 is required for cell elongation and osmoregulation process [15, 60]. Its involvement in osmotic adjustment may explain the slight increase in *OsCCC1* expression in plants exposed to PEG.

Gene coding for CLC1 displayed a similar pattern of expression than gene coding for CCC1: it was moderately stimulated in response to NaCl after 1 day of exposure, but strongly induced after 7 days. CLC1 is supposed to act as a voltage-gated chloride channel located at the tonoplast and is involved in Cl^-^ vacuolar sequestration [61]. According to Diédhiou and Golldack [62], *OsCLC1* expression in *O*. *sativa* was stimulated in roots and shoots of a salt-tolerant cultivar (Pokkali) while it was repressed in a salt-sensitive one (IR29). Such difference was not recorded in our study on *O*. *glaberrima* and both TOG5307 and TOG5949 had almost similar levels of expression. It has to be mentioned that CLC may also be involved in NO_3_^-^ nutrition [19] and, according to De Angeli et al. [63], Cl^-^ transport may even be much less than NO_3_^-^. Gene coding for CLC1 was also up-regulated in response to PEG and Li et al. [64] reported that this gene may be induced by dehydration, suggesting its possible role in osmoregulation through NO_3_^-^ accumulation besides its role in Cl^-^ vacuolar sequestration. This might explain the high expression recorded in the shoot of salt-tolerant cultivar TOG5307 after 1 day and in the roots of both cultivars after 7 days of exposure to PEG. Similarly, OsNRT1 is a low Cl^-^ transporter [17]. It has however to be mentioned that this poorly selective transporter also transports NO_3_^-^ anions through plasma membrane [20] so that its stimulation may also reflect the need to supply nitrogen to stressed plants rather than an attempt to regulate osmotic potential by Cl^-^ influx; the same gene was also overexpressed at the shoot level and in both cultivars after 7 days.

### Ion accumulation and gene expression in the MIX treatment

The simultaneous presence of NaCl and PEG allowed us to quantify the impact of non-ionic osmotic stress on ion accumulation and expression of genes coding for transporters. It was expected that, when a significant difference did occur between PEG and NaCl treatments, plants exposed to the MIX treatment should exhibit intermediate behavior. This is however far from being the case. Indeed, chloride and sodium accumulations were strongly reduced in the shoots when PEG was present. Even if the exogenous dose of NaCl used in the MIX treatment was reduced by 50%, the level of accumulation in response to MIX treatment was unexpectedly low (and even in some case similar to control) and this could not be explained by stomatal closure or by a decrease in transpiration rates. PEG is known to induce an increase in the viscosity of nutrient solution, but it is hard to believe that this process may alter Na^+^ and Cl^-^ diffusion to such an extent that it compromises ion absorption by the roots, especially considering that PEG-treated plants did not suffer from K^+^ deficiencies. Hence, these observations suggest that the non-ionic water stress may drastically alter accumulation of toxic ions, although this did not lead to any significant improvement of plant growth or water status. Another unexpected (and still unexplained) result is that even if Na^+^ accumulation was quite lower in MIX-treated plants than in NaCl-exposed ones, the decrease in K^+^ concentration was almost similar for the two treatments (Fig 1c). Despite the strong difference in Cl^-^ accumulation between NaCl and MIX treatment, Cl^-^ transporters genes were regulated exactly in the same way in the two treatments, especially after 7 days as noticed for *OsCCC1*, *OcCLC* and, to a lower extent, for *OsNRT1* (Fig 2). In contrast, some genes involved in Na^+^ transport exhibited a lower expression in response to MIX treatment than in NaCl-treated plants. This was the case for *OsSOS1* and *OsSOS2*: since those genes are involved in Na^+^ extrusion, their lower expression in MIX-treated plants than in NaCl-exposed ones cannot fully explain the lower accumulation of Na^+^ in the root of the former comparatively to the latter.

Additional molecular studies focusing on involved transcription factors and transduction signals are required to explain these unexpected data.

## Supporting information

S1 Table Statistical effects of cultivar (C), treatment (T), duration (D) and their interactions as determined by 3-way analysis of variance for parameters recorded on African rice seedlings (*Oryza glaberrima* Steud.) from salt-resistant (TOG5307) and salt-sensitive (TOG5949) cultivars. Symbols: *, **, *** and **** represent statistical significance at *P* < 0.05, 0.01, 0.001 and 0.0001, respectively; and NS = not significant at *P* = 0.05. Data related to growth parameters were considered for 7 days of treatment, only.
(DOCX)
