# Supplementary material for: Comparison between the impact of osmotic and NaCl treatments on the expression of genes coding for ion transporters in Oryza glaberrima Steud
Source: PLoS One. 2023 Nov 15;18(11):e0290752. doi: 10.1371/journal.pone.0290752 (PMC10650995; doi:10.1371/journal.pone.0290752)
Supplement: S1 Table — Symbols: *, **, *** and **** represent statistical significance at P < 0.05, 0.01, 0.001 and 0.0001, respectively; and NS = not significant at P = 0.05. Data related to growth parameters were considered for 7 days of treatment, only. (DOCX) [file pone.0290752.s001.docx]

**Table S1.** Statistical effects of cultivar (C), treatment (T), duration (D) and their interactions as determined by 3-way analysis of variance for parameters recorded on African rice seedlings (*Oryza glaberrima* Steud.) from salt-resistant (TOG5307) and salt-sensitive (TOG5949) cultivars. Symbols: *, **, *** and **** represent statistical significance at *P* < 0.05, 0.01, 0.001 and 0.0001, respectively; and NS = not significant at *P* = 0.05. Data related to growth parameters were considered for 7 days of treatment, only.

| Parameters | | Analyse of Variance (ANOVA 3) | | | | | | |
| --- | --- | --- | --- | --- | --- | --- | --- | --- |
|  |  | C | T | D | CxT | CxD | TxD | CxTxD |
| Relative incraese in the leaf number | | *** | NS | - | NS | - | - | - |
| *F_v_*/*F_m_* | | **** | **** | **** | NS | NS | **** | *** |
| Ф_PSII_ | | **** | **** | **** | * | NS | * | NS |
| qP | | *** | **** | **** | * | NS | **** | NS |
| NPQ | | **** | **** | **** | **** | ** | **** | * |
| *A* | | **** | **** | *** | **** | *** | **** | NS |
| *E* | | **** | **** | **** | * | NS | **** | NS |
| *g*_s_ | | **** | **** | **** | **** | **** | **** | **** |
| MDA | | **** | **** | **** | **** | **** | **** | **** |
| Proline | | **** | **** | **** | ** | *** | **** | * |
| Total Sugars | | **** | **** | **** | NS | NS | **** | NS |
| *Ψ*s (MPa) | Root | ** | **** | * | NS | NS | * | NS |
|  | Shoot | **** | **** | **** | **** | **** | **** | NS |
| Sodium | Root | **** | **** | NS | **** | ** | ** | NS |
|  | Shoot | **** | **** | **** | **** | NS | **** | NS |
| Potassium | Root | NS | **** | **** | * | NS | **** | NS |
|  | Shoot | NS | **** | **** | NS | NS | **** | NS |
| Na/K ratio | Root | *** | **** | NS | **** | NS | NS | NS |
|  | Shoot | **** | **** | NS | **** | NS | **** | NS |
| Chloride | Root | **** | **** | NS | **** | ** | ** | NS |
|  | Shoot | **** | **** | **** | **** | NS | **** | NS |
| Relative elongation | Root | **** | **** | - | *** | - | - | - |
|  | Shoot | **** | **** | - | **** | - | - | - |
| Fresh Weight | Root | **** | **** | - | * | - | - | - |
|  | Shoot | **** | **** | - | NS | - | - | - |
| Dry Weight | Root | *** | **** | - | NS | - | - | - |
|  | Shoot | ** | **** | - | NS | - | - | - |
| Water content | Root | NS | * | - | NS | - | - | - |
|  | Shoot | NS | **** | - | *** | - | - | - |
| *OsHKT1.5* | Root | **** | **** | **** | **** | **** | **** | * |
|  | Shoot | **** | **** | **** | **** | *** | **** | NS |
| *OsHKT1.1* | Root | - | - | - | - | - | - | - |
|  | Shoot | **** | **** | **** | *** | * | **** | ** |
| *OsNHX1* | Root | **** | **** | **** | **** | NS | **** | * |
|  | Shoot | **** | **** | **** | **** | NS | **** | NS |
| *OsNHX2* | Root | **** | **** | **** | **** | NS | **** | NS |
|  | Shoot | *** | **** | **** | *** | NS | **** | ** |
| *OsSOS1* | Root | **** | **** | **** | *** | NS | **** | NS |
|  | Shoot | **** | **** | **** | *** | NS | **** | * |
| *OsSOS2* | Root | * | **** | - | NS | - | - | - |
|  | Shoot | NS | **** | - | NS | - | - | - |
| *OsNRT1* | Root | *** | **** | **** | ** | * | **** | ** |
|  | Shoot | **** | **** | **** | **** | NS | **** | NS |
| *OsCLC* | Root | NS | **** | **** | **** | NS | **** | * |
|  | Shoot | NS | **** | **** | **** | NS | **** | NS |
| *OsCCC1* | Root | **** | **** | **** | **** | **** | **** | NS |
|  | Shoot | **** | **** | **** | **** | ** | *** | NS |
| *OsDREB2* | Root | ** | **** | **** | NS | NS | **** | NS |
|  | Shoot | **** | **** | **** | **** | * | *** | NS |
